# Are Exposures to Multiple Frequencies the Key to Future Radiofrequency Research?

**DOI:** 10.3389/fpubh.2017.00328

**Published:** 2017-12-08

**Authors:** Zenon Sienkiewicz, Carolina Calderón, Kerry A. Broom, Darren Addison, Amélie Gavard, Louise Lundberg, Myron Maslanyj

**Affiliations:** ^1^Centre for Radiation, Chemical and Environmental Hazards, Public Health England, Chilton, United Kingdom

**Keywords:** multiple exposures, cancer, animal studies, radiofrequencies, review

## Abstract

There is an extensive literature investigating possible effects of exposure to radiofrequency (RF) electromagnetic fields associated with mobile phone technologies. This has not identified any public health risks with any degree of certainty. Some epidemiological studies have observed associations between heavy users of mobile phones and some types of cancer, but animal studies do not support this association, although a few studies have reported increased tumor yields. However, there is a crucial difference between epidemiology studies and laboratory work in terms of signals investigated: most people are exposed to a complex mixture of frequencies and signals at varying intensities, whereas the majority of animal studies have been performed using a single frequency or intensity. Whether this might explain the differences in outcome will be discussed, and whether there is a need for additional laboratory investigations that reproduce more accurately realistic exposure conditions will be considered.

## Introduction

Around the turn of this century, concerns about mobile phone technology prompted many countries to instigate scientific research programmes into possible health effects of low-level exposure to radiofrequency (RF) electromagnetic fields. These included the Mobile Telecommunications and Health Research Programme in the UK, the German Mobile Communication Research Programme, and the Fondation Santé et Radiofréquences in France. The endpoints studied reflected public concerns at the time, and included risks of childhood and adult cancers, and effects on nervous system function. The possibility that some individuals experience hypersensitivity or non-specific symptoms in response to exposure was also considered a high priority for research. Uncertainties about possible health effects also encouraged many additional studies outside of these research programmes, including EU-funded projects, such as EMF-NET, EFHRAN, and Mobi-Kids, and this has resulted in a very extensive literature.

The results of all these studies have not identified any public health risks with any degree of certainty. Some epidemiological studies have observed associations between heavy users of mobile phones and some types of cancer, but few animal cancer or mechanistic studies provide support for this association (1–3). Associations with other diseases and endpoints are less well established.

There is a crucial difference between epidemiology studies and laboratory work in terms of signals investigated: most people are exposed to a complex mixture of frequencies and signals at varying intensities (i.e., not just the RF signals from a mobile phone but also additional RF, and lower frequency fields), whereas the majority of animal studies have been performed using a single frequency, and often a signal from a second (or more recently, a third) generation mobile phone. Thus, it might be argued that there is a need for additional laboratory investigations that reproduce more accurately typical exposure conditions.

This paper summarizes the typical exposures that members of the general public may experience on an everyday basis, reviews the experiments investigating carcinogenesis that have been performed with animals exposed to single or multiple RF electromagnetic fields, and considers whether there are gaps in knowledge to suggest possible future research needs. As yet, no laboratory studies investigating potential carcinogenic effects have been performed using exposures that include contributions of sources both close-to and far-from the body that may be typically experienced by a member of the public.

## Summary of Typical Public Exposures

Members of the public are exposed to many electromagnetic fields from a large number of sources and over a large range of field intensities and frequencies. For instance, in the home, in addition to localized exposures from mobile phones, people may be exposed simultaneously to the RF signals from wireless networks (Wi-Fi), smart meters for monitoring of domestic energy usage and mobile phone base stations (downlink signals). At the same time, intermediate frequency signals are produced by appliances such as induction hobs and various lighting equipment and the electricity supply, while wireless communication devices and various electrical appliances produce electric and magnetic fields at power frequencies (but these applications are not considered further here).

Examples of exposures to RF electromagnetic fields that may occur in a typical public environment are shown in Table 1 (uniform exposure of the whole body) and Table 2 (localized exposure of the head). Comprehensive studies have been carried out by Rowley and Joyner (4) and Gajšek and colleagues (5). These authors and others have highlighted the limitations of quantifying exposure using personal exposure, which may be subject to large uncertainties including the effect of body shielding (4–6), while narrowband measurements may be less representative of average personal exposures as they tend to be performed outside and close-to base stations (4, 5, 7).

**Table 1:** Examples of typical uniform radiofrequency exposures and sources likely to be encountered by a member of the public from personal dosimetry data.

Frequency range, MHz	Source	Waveform	Electric field (RMS), mV/m		Equivalent Whole-body specific energy absorption rate (SAR), μW/kg		Number of samples		Reference	
			
			Urban	Rural	Urban	Rural	Urban	Rural	Urban	Rural
88–108	FM	FM	19–190	39–55	0.81–7.95	1.63–2.3	795	30	(8)	(9)
174–223	DAB/TV	OFDM/QPSK	19–183	–	0.40–3.75	–		–		–
470–830	TV	OFDM/QPSK	19–183	43–48	0.32–3.05	0.72–0.80		30		(9)
370–400	Tetrapol	FDMA	0.00–27	–	0.00–0.56	–		–		–

791–821	LTE 800 Downlink	OFDM	0.00	27–34	0.00	0.46–0.57
925–960	Downlink 900	TDMA	159–307	133–143	3.19–4.77	2.26–2.43
1,805–1,880	Downlink 1,800	TDMA	208–311	136–143	3.19–4.77	2.09–2.19	20	30	(9)
1,880–1,900	DECT	TDMA	27–43	19	0.41–0.65	0.29
2,110–2,170	Downlink 2,100	WCDMA	134–152	80–93	1.88–2.13	1.12–1.30
2,620–2,690	LTE 2,600 Downlink	OFDM	0.00	0.00	0.00	0.00

2,400–2,485	Smart meters	DSSS, OQPSK	87–215		0.01–0.30^a^		39		(10)
2,401–2,485	Wi-Fi	OFDM	0.00–19	0.00	0.14	0.00	20	30	(9)
5,150–5,872	Wi-Fi	OFDM	27	27	0.35	0.31	20	30

*Measurements were performed with a personal exposure meter (DSP120 EME SPY (SATIMO) or ExpoM-RF) except for smart meters where measurements under controlled laboratory conditions were made with a Q-par Angus QSH12N10S horn antenna connected to an Agilent N9020A MXA signal analyzer. Equivalent whole-body specific energy absorption rate (SAR) values were calculated using conversion factors from Ref. (11) below 3 GHz and (12) at 5 GHz. Note the latter used the NORMAN phantom scaled to a 10-year-old. OFDM, Orthogonal Frequency-Division Multiplexing; QPSK, Quadrature Phase Shift Keying; FDMA, Frequency Division Multiple Access; TDMA, Time Division Multiple Access; WCDMA, Wideband CDMA; DSSS, Direct Sequence Spread Spectrum*.

*^a^Laboratory measurements made at 1.5 m in direction of maximum electric field strength. Smart meter devices have a duty factor of around 1% (10); this has been included in the SAR value only. Unless specified, all measurements include duty factors. The range of values within each study is shown*.

**Table 2:** Summary of localized radiofrequency exposures and sources likely to be encountered by a member of the public.

Frequency range, MHz	Source	Specific energy absorption rate (SAR) 10 g head, mW/kg	Waveform	Number of samples	Comment	Reference
880–915	GSM	268 (15–557)	TDMA	351	Values have been scaled by 0.5 to account for adaptive power control (typical power levels) and a further 0.7 to account for Discontinuous Transmission (13)	(13)
1,710–1,785	GSM	180 (14–595)	TDMA	120	
1,920–1,980	UMTS	5 (0.4–18)	WCDMA	120	Estimated using SAR from GSM1800 and scaling it to take into account differences in duty factor (1:1 versus 1:8), maximum output power (125 mW versus 1 W) and typical power levels (1% of maximum versus 50%). Does not take into account difference in frequency
1,880–1,900	DECT phones	45 (3–148)	TDMA	120	Estimated using SAR from GSM1800 and scaling it to take into account differences in duty factor (1:24) and output power (0.250 W)
2,400–2,484	Wi-Fi laptops	2 (1.0–3.3)	OFDM	15	Estimated using average Equivalent Isotropically Radiated Power from lab measurements (14) and scaling factors for SAR modeling of an inverted F-antenna in a laptop placed 34 cm from a scaled 10-year-old (12). Measurements in schools suggest that duty factor of laptops during classrooms is around 0.08%, *N* = 146 (15), while another study found a median of 1.4% for various environments and activities, *N* = 179 (16)	(12, 14)
5,150–5,872		2 (0.4–3.8)	OFDM	8		

*All exposures are for handheld phones during voice calls. Values are arithmetic means and ranges of values are shown in brackets. Values do not include duty factor from typical usage but takes into account duty factor from TDMA communication systems (i.e., 1:8 for GSM and 1:24 for DECT)*.

*GSM, Global System for Mobile communication; UMTS, Universal Mobile Telecommunications Service; DECT, Digital Enhanced Cordless Telecommunications; TDMA, Time Division Multiple Access; WCDMA, Wideband Code Division Multiple Access; OFDM, Orthogonal Frequency-Division Multiplexing*.

In general, exposure levels are influenced by the power output of the device, its proximity to the body, and the duration of exposure. In addition, it is usually necessary to take into account the extent of the exposure, for instance, a local source such as a mobile phone held to the head produces a very localized exposure at the head whereas a signal from a distant mobile phone base station produces a more uniform exposure over the extent of the body. An ongoing challenge is how to translate this complex pattern of emissions into an integrated measure of exposure for use in epidemiology (17, 18).

Overall, a person can be exposed to a large mixture of RF and other electromagnetic fields at any one time depending on where they are and these exposures also vary in time.

## Summary of Epidemiological Results

A large number of epidemiological studies of mobile phone use and cancer risk have been carried out. Interest has focused on tumors of the head and neck region because these are the tissues that are primarily exposed by the RF fields from mobile phones, but some studies have investigated other types of tumors, including leukemia, lymphoma, and malignant melanoma. Additional studies have investigated effects of occupational exposure to RF fields, or examined time-trend data for brain tumors. All these data have been extensively studied and reviewed by many expert groups, including the independent Advisory Group on Non-ionizing Radiation (1), the International Agency for Research on Cancer (IARC) (2) and the Scientific Committee on Emerging and Newly Identified Health Risks (SCENIHR) (3) and only a brief summary of the overall conclusions is presented here.

Taken together, the epidemiological studies do not provide consistent evidence of a carcinogenic effect of RF exposure at levels encountered in the general population. Nevertheless, IARC concluded that there was limited evidence in humans for the carcinogenicity of RF fields, due to the positive associations observed between glioma and acoustic neuroma and exposure to RF fields from wireless phones, and overall RF fields were considered to be possibly carcinogenic to humans (2). This means that a causal link between RF fields and an increased risk of cancer was considered to be credible, but some combination of chance, bias or confounding in the data could not be ruled out with an acceptable degree of confidence.

The bulk of evidence for this decision from IARC (2) came from the INTERPHONE study and a Swedish case–control study by Hardell and colleagues. In particular, the INTERPHONE study did not observe raised risks of brain tumors, acoustic neuroma, or parotid gland tumors among regular mobile phone users, and the risk estimates did not increase with longer time since first mobile phone use. There were, however, suggestions of an increased risk of glioma at the highest exposure level (1640 h or more of cumulative phone use) and among long-term users in the most exposed areas of the brain (19, 20). It is possible that exposure may have caused modest risk increases in only the heaviest users of mobile phones. If correct, such increases may not be detectable in cohort studies or in the time-trend data (3). Exposure misclassification is a considerable problem in such studies, and in relation to the present discussion, exposures from other sources are rarely taken into account to any meaningful degree. Based on cohort and case–control studies published since the IARC assessment, SCENIHR concluded that the evidence for glioma had become weaker (3). However, SCENIHR acknowledged that research was still lacking in some areas, particularly for investigating long-term effects associated with mobile phone use, and recommended prospective cohort studies in adults and children as a high priority.

Overall, the evidence from epidemiology studies does not suggest that exposure to RF fields associated with mobile phone use is a significant health risk for most people. However, the possibility that increased risks may exist in the small number of very high users cannot be excluded with certainty, although these increased risks may be related to previous technology that tended to expose the tissues of the head and neck to more intense fields. The retrospective assessment of exposures from mobile phones remains an ongoing challenge given the rapidly changing technologies and changes in behavioral pattern of use.

## Summary of Studies with Animals

Excluding any study that may have obvious shortcomings in methodology or had insufficient or absent dosimetry, such as using an unmodified mobile phone as an exposure source (3), most recent long-term animal studies using a single frequency have reported a lack of carcinogenic effects in a variety of animal models, including classical long-term bioassays (Table 3), studies using transgenic and tumor-prone animals (Table 4), co-carcinogenicity studies involving combined exposure to RF fields and known carcinogens (Tables 5 and 6), and studies evaluating effects on the development of tumors after transplantation or inoculation of tumor cells (Table 7). Generally, these studies used frequencies associated with mainstream mobile technology, and assessed tumor yields using post-mortem pathology, although the studies included in Table 7 used very high frequencies (40–60 GHz) and at field strengths that caused significant increases in local tissue temperatures, which are less representative of exposures from mobile phones. All these studies were judged and selected for inclusion in the tables using quality criteria similar to those used in a systematic review of phone use and brain cancer (21). These criteria included having adequate study size, use of an appropriate exposure system, and suitable data analysis.

**Table 3:** Animal studies investigating carcinogenic potential of radiofrequency fields in conventional strains.

Endpoint	Exposure conditions	Results of exposure	Comments	Reference
Tumors in male and female Fischer rats	835 MHz FDMA, or 847 MHz CDMA; 4h/day, 5 days/week for 2 years; at 1.3 ± 0.25 W/kg in brain, animals restrained	No significant effects on survival, growth, incidences of brain tumors or other neoplasms, or on non-neoplastic lesions	Restraint devices increased in size with animals’ age. Animals irradiated in early morning	(22)
Brain tumors in male and female Fischer 344 rats	Iridium 1.6 GHz; 2 h/day from gd 19 until postnatal day 23 ± 2 (weaning), at 0.16 W/kg in brain, animals freely moving; and 2 h/day, 5 days/week from 35 days old until 2 years of age, at 0.16 or 1.6 W/kg in brain, head-only exposure, animals restrained	No significant effect on early survival, weaning or growth weights, clinical signs, or incidences of brain tumors or other neoplasms	Significant increase in weight in male and female cage controls, and significant decrease in survival of female cage controls	(23)
Tumors in male and female Han Wistar rats	GSM 902 MHz or DCS 1747 MHz; 2 h/day, 5 days/week for 2 years; at 0.4, 1.2, or 3.7 W/kg GSM; or 0.4, 1.3 or 4 W/kg DCS; animals restrained	Some incidental differences, but no significant effects on health status, clinical signs, food consumption, body or organ weights, or mortality. No significant increases in numbers of tumor-bearing animals, total numbers of tumors, or in any specific tumor type	Specific energy absorption rate (SAR) of GSM reduced due to large rat growth. Exposure consisted of three different 40-min phases emulating talking, listening, and moving in environment. Highest exposure below thermal threshold. Carried out under good laboratory practice standards	(24)
Tumors in male and female B6C3F1 mice	GSM 902 MHz or DCS 1747 MHz; 2 h/day, 5 days/week for 2 years; at 0.4, 1.3, or 4 W/kg; animals restrained	No significant effects on health status, clinical signs, food consumption, body or organ weights, or mortality. No significant increases in numbers of tumor-bearing animals, total numbers of tumors, or in any specific tumor type	Exposure consisted of three different 40-min phases emulating talking, listening, and moving in environment. Highest exposure below thermal threshold. Incidence of all tumor types in line with historical values. Carried out under good laboratory practice standards	(25)
Tumors, in female SD rats	GSM 900 MHz; continuous exposure, except for 15 min/day (feeding), 4 × 1–2 h/week (health check and cleaning), 4–5 h/month (servicing) for up to 3 years of age; at 0.08 W/kg (young) to 0.038 W/kg (old); group of 12 animals freely moving in home cage	No significant effects on incidence of pituitary, mammary, or other tumors weight gain or survival with exposures <2 years. Significant reduction in median survival with exposures lasting >2 years	Modest group sizes. Effects on survival modulated by time of year of birth	(26)

*SD, Sprague-Dawley; FDMA, Frequency Division Multiple Access; TDMA, Time Division Multiple Access; GSM, Global System for Mobile communication; DCS, Digital Cellular System; gd, gestational day*.

*SAR values are mean whole-body averages unless indicated otherwise; significant indicates statistical significance*.

**Table 4:** Animal studies investigating the potential of radiofrequency fields to promote tumors in tumor-prone animals.

Endpoint	Exposure conditions	Results of exposure	Comments	Reference
Lymphoma in AKR/J mice, analysis of blood	900 MHz GSM, 24 h/day for 41 weeks, at 0.4 W/kg, in home cage, animals freely moving	No significant effects on survival, incidence of lymphoma, blood cell counts. Significant increase in weight gain	Field turned off for 1 h twice per week for cleaning, animal inspection	(27)
Lymphoma in AKR/J mice, analysis of blood	UMTS test signal, 1.966 GHz, 24 h/day for 35 weeks, at 0.4 W/kg, in home cage, animals freely moving	No significant effects on survival, incidence of lymphoma, lymphatic infiltrations, white blood cell counts, weight gain. Lower weight in cage controls attributed to different feeding methods	Field turned off for 1 h twice per week for cleaning, animal inspection	(28)
Lymphoma in *Pim1* transgenic mice	900 MHz, pulse width 0.577 ms, 217 Hz, 1 h/day for 18 months, at 0.5, 1.4, or 4 W/kg, animals restrained	Sporadic changes, but no consistent effects on clinical signs, weight gain, incidence of lymphoma, histiocystic sarcoma, or other tumors. Survival decreased in all groups of males, and in females at 0.5 W/kg	Includes animals at end of exposure. Significant differences in cage control animals. Carried out under good laboratory practice standards	(29)
Multiple tumors (medulloblastoma, rhabdomyosarcomas, or preneoplastic lesions typical of basal cell carcinomas) in *Patched1 (Ptc1)* heterozygous mice	900 MHz GSM, 2 × 30 min/day for 5 days, from postnatal day 2–6, at 0.4 W/kg, animals restrained in polystyrene jigs	No significant decrease in survival, no significant increase in incidence, onset or histology of tumors, or in preneoplastic skin lesions. No effects on liver or other neoplasms	*Ptc1* show peak sensitivity to X-rays during early postnatal life	(30)

*GSM, Global System for Mobile communication; UMTS, Universal Mobile Telecommunications Service*.

*Specific energy absorption rate values are mean whole-body averages; significant indicates statistical significance*.

**Table 5:** Animal studies investigating co-carcinogenic effects of radiofrequency fields following transplacental ENU administration.

Endpoint	Exposure conditions	Results of exposure	Comments	Reference
CNS tumors in male and female Fischer 344 rats	836.55 MHz FM talk signal; 2 h/day from dg 19 until postnatal day 21, animals freely moving; and 2 h/day, 4/days week, from day 31 for 2 years, at 1–1.2 W/kg in brain, animals restrained; and/or single maternal intravenous injection of ENU (4 mg/kg on dg 18)	No significant effects on survival, number, incidence, or any tumor type	Effects with ENU. No effect on spontaneous tumors	(31)
CNS tumors in SD rats	860 MHz pulsed or continuous wave MiRS signal, 6 h/day, 5 days/week from 53 days old to 24 months, at 1 W/kg in brain, animals restrained, and/or single maternal intravenous injection of ENU (2, 5, or 10 mg/kg on dg 15)	No significant effects on brain or spinal cord tumors	Carried out under good laboratory practice standards	(32)
CNS tumors in Fischer 344 rats	1.439 GHz TDMA signal, 90 min/day, 5 days/week from 5 weeks of age, for 104 weeks at 0.67 or 2 W/kg in brain, head-only exposure, animals restrained, and/or single maternal intravenous injection of ENU (4 mg/kg on dg 18)	No significant effects on CNS tumors, pituitary tumors significantly reduced in males at 2 W/kg, no significant effect on growth or survival	Carried out under good laboratory practice standards	(33)
CNS tumors in SD rats, assessed every 30 days 171 to 325 days	860 MHz pulsed, MiRS signal, 6 h/day, 5 days/week (excluding holidays) from 50 days old, at 1 W/kg in brain, animals restrained, and/or single maternal intravenous injection of ENU (6.2 or 10 mg/kg on dg 15)	No significant effects on incidence, malignancy multiplicity or latency of spinal cord or spinal nerve tumors, cranial nerve tumors, or brain tumors		(34)
CNS tumors in Fischer 344 rats	1.95 GHz WCDMA signal, 90 min/day, 5 days/week from 5 weeks of age, for 104 weeks at 0.67 or 2 W/kg in brain, head-only exposure, animals restrained, and/or single maternal intravenous injection of ENU (4 mg/kg on dg 18)	No significant effects on CNS tumors, skin fibromas and large granular lymphocytic leukemia significantly reduced in males exposed at 2 W/kg, no significant effect on growth or survival	Carried out under good laboratory practice standards	(35)
Tumors in B6C3F1 female mice	1.966 GHz UMTS, 20 h/day for up to 24 months starting on dg 6, at 4.8 or 48 W/m, peak specific energy absorption rate (SAR) calculated at 5 W/kg and/or single maternal intraperitoneal injection of ENU (40 mg/kg) on dg 14 in low exposure group, animals freely moving	No significant effects with UMTS alone. Incidence, malignancy, and multiplicity of lung carcinomas significantly increased in ENU + UMTS, and numbers of lung metastases (non-significantly) doubled. Effects on liver tumors discounted due to *Helicobacter* infection	Highest SAR did not induce increase in temperature	(36)
Tumors in B6C3F1 female mice	1.966 GHz UMTS, 23.5 h/day for 72 weeks starting on dg 6, at 0.04, 0.4, or 2 W/kg and single maternal ip injection of ENU (40 mg/kg) on dg 14, animals freely moving	Significant increases in lung adenomas and liver carcinomas at all SARs, lung carcinoma, and lymphomas at 0.4 W/kg. No dose–response. No increase in any tumor in brain, kidney, spleen	No UMTS-only group. No *Helicobacter* infection at 1 year	(37)

*CNS, central nervous system; ENU, N-ethyl-N-nitrosourea; SD, Sprague-Dawley; MiRS, Motorola integrated Radio Services; TDMA, Time Division Multiple Access; WCDMA, Wideband Code Division Multiple Access; UMTS, Universal Mobile Telecommunications Service; dg, gestational day; ip, intraperitoneal*.

*SAR values are mean whole-body averages, unless indicated otherwise; significant indicates statistical significance*.

**Table 6:** Animal studies investigating co-carcinogenic effects of radiofrequency fields with other carcinogenic agents.

Endpoint	Exposure conditions	Results of exposure	Comments	Reference
Mammary tumors in female SD rats, following initiation with DMBA	GSM 900 MHz; continuous exposure, except for 10–20 min/day (feeding), 3 × 1–2 h/week (cleaning), 3–4 h/week for tumor palpation, 4–5 h/month (servicing) for up to 334 days; at 0.03–0.13 W/kg (young) to 0.01–0.06 W/kg (old); group of 12 animals freely moving, and DMBA (50 mg/kg) by gavage	Overall, no significant effects on incidence or latency of benign or malignant tumors. Incidence of malignant tumors significantly reduced only in first experiment	Three experiments in total. Exposures began in evening after DMBA treatment. Animals sacrificed when tumors were 1–2 cm in diameter	(38)
Skin tumors in male ICR mice, following initiation with DMBA, assessed at sacrifice after 20 weeks	848.5 or 1762 MHz CDMA, 2 × 45 min/day, 5 days/week for 19 weeks, at 0.4 W/kg, animals freely moving, and DMBA (100 µg per 100 µl) painted on dorsal skin	No skin tumors, and no effects on epidermis	Exposures began 7 days after DMBA treatment, each 45-min exposure separated by 15 min. Significant effects seen with phorbol acetate	(39)
Tumors in female Wistar rats, with MX throughout study	900 MHz GSM, 2 h/day, 5 days/week for 104 weeks, at 0.3 or 0.9 W/kg, animals freely moving, and MX (1.7 mg/kg in drinking water)	No significant effects on organ-specific incidence of any tumor type, effect in merged vascular tumors attributed to chance		(40)
Mammary tumors in female SD rats, following single initiation with DMBA	900 MHz GSM, 4 h/day, 5 days/week for 26 weeks at 0.44, 1.33 or 4 W/kg, animals restrained, and DMBA (35 mg/kg) by gavage	No significant effects on benign or malignant mammary tumors	Exposures began 1 day after DMBA treatment. Significant differences in weight, tumor incidence, and latency in cage controls	(41)
Mammary tumors in female SD rats, following initiation with DMBA	902 MHz GSM, 4 h/day, 5 days/week for 186 days at 0.44, 1.33 or 4 W/kg, animals restrained, and DMBA (17 mg/kg) by gavage	Sporadic significant differences observed, but no dose-related trends. Overall, no differences attributed to exposure	Exposures began 1 day after DMBA treatment. Significant differences in tumor incidence and malignancy in cage controls carried out under good laboratory practice standards	(42)

*SD, Sprague-Dawley; CDMA, Code Division Multiple Access; GSM, Global System for Mobile communication; MX, 3-chloro-4-(dichloromethyl)-5-hydroxy-2(5H) furanone: DMBA, 7,12-dimethylbenz(a)anthracene*.

*Specific energy absorption rate values are mean whole-body averages, unless indicated otherwise; significant indicates statistical significance*.

**Table 7:** Animal studies investigating effects of radiofrequency fields on implanted tumor cells.

Endpoint	Exposure conditions	Results of exposure	Comments	Reference
B16 F10 melanoma cells injected sc into Swiss Webster mice	61.22 GHz, 15 min/day for 5 days at 133 W/m to the head, animals restrained	Significantly reduced tumor growth with exposures starting on day 5 after injection, no significant effects with exposures starting on day 1 or 10	Effect blocked by naloxone hydrobromide (1 mg/kg). Maximum temperature rise of around 1°C at tip of nose	(43)
B16 F10 melanoma cells injected sc into SKH1 hairless mice, and/or CPA (30 or 20 mg/kg) injected ip, on days 4–8	42.2 GHz, 60 Hz modulation, 30 min/day for 5 days at 365 W/m (peak) to the nose [peak specific energy absorption rate (SAR) of 730 W/kg], animals restrained	Dose-dependent reduction in tumor growth with CPA, no additional effects with exposure on days 4–8 post-inoculation, nor with exposure before and/or after CPA	Temperature rise of 1.5°C on the nose	(44)
B16 F10 melanoma cells injected intravenously in female C57BL/6 mice on day 2 post-exposure, and/or CPA (150 mg/kg) ip; numbers of metastatic lung colonies counted after 2 weeks	42.2 GHz, 60 Hz modulation, 30 min at 365 W/m (peak) to the nose (peak SAR of 730 W/kg), animals anesthetized	CPA alone significantly increased metastases, RF alone or RF + CPA significantly decreased metastases, RF + CPA significantly increased activity of natural killer cells	Temperature rise of 1.5°C on the nose	(45)

*sc, subcutaneous; ip, intraperitoneal; CPA, cyclophosphamide*.

*Significant indicates statistical significance*.

However, it is of interest that one study (36) reported positive results in a pilot study using transplacental administration of ethylnitrosourea (ENU) to induce mutagenicity in brain tissues. It was found that lifetime exposure to 1966 MHz UMTS signals at a whole-body specific energy absorption rate (SAR) of up to 5 W/kg (peak) had no effect on incidence of brain tumors, but exposure increased the incidence and multiplicity of lung carcinoma in female mice compared with animals treated with ENU alone. Significant effects were also seen on liver tumors, but these were discounted due to possible confounding caused by bacterial infection. UMTS exposure on its own had no tumorigenic effect.

These results were independently replicated using the same experimental design although the study was improved by using a larger number of exposure groups (37). Animals were exposed at a whole-body average SAR of up to 2 W/kg. Generally, the results of this second study were consistent (but not identical) with those of the pilot study, possibly indicating the inherent variability in tumor incidence with this model. Prenatal ENU treatment has been considered an ideal experimental model for the study of brain tumors in transgenic mice (46) so the absence of any increase in brain tumors in both studies is intriguing, but could be due to factors such as the dose of ENU used, the time at which it was injected, or strain-specific sensitivity. In addition, a dose–response was absent in the replicate study with no explanation offered as to why exposure at 0.4 W/kg (but not at 0.04 or 2 W/kg) should have had the most consistent effect on tumor promotion.

In addition, the much-anticipated first report from the National Toxicology Program (NTP) study on the effects of RF fields on rodent carcinogenesis has been published online (47). This suggested that lifetime, intermittent exposure to CDMA or GSM 900 MHz signals at a whole-body average SAR of 1.5–6 W/kg for 18 h per day increased the incidence of malignant gliomas in the brain and schwannomas of the heart in male, but not female, rats. Results from other organs have not yet been reported, nor any results from the other half of the study using mice. Overall, the available results are far from conclusive, not least because many pertinent details about the study are missing from the report and concerns exist about the unexpected absence of tumors in the (single) control group. Comments made by one of the reviewers of the study which were included as an appendix in the report indicate that if the tumor incidence rate in the control group had occurred at the historical rate for those rats in that institution, then the results would not have reached statistical significance. Comprehensive analysis on this study must await full publication of the complete NTP dataset. (This study is not included in Table 3 for these reasons.)

Together, the pilot study (36) and replicate study (37) using the transplacental ENU model perhaps provide the strongest experimental evidence to suggest that long-term exposure to single RF fields may have some tumor promoting effects, but the evidence remains inadequate to make definitive conclusions. This is due in part to differences in results between the two studies, the lack of an obvious dose–response relationship (37), and unresolved questions about the potential nature of the biological interaction mechanism. Importantly, other studies using similar models have not reported comparable effects (Table 5).

Only two studies appear to have investigated the direct carcinogenic effects of combined exposure to more than one RF field (Table 8). In both studies, which come from the same research group, unrestrained animals were exposed using a reverberation chamber to 849 MHz CDMA and 1950 MHz WCDMA signals at a whole-body average SAR of 2 W/kg/signal (making 4 W/kg in total).

**Table 8:** Animal studies investigating carcinogenic potential of simultaneous exposure to two different RF frequencies.

Endpoint	Exposure conditions	Results of exposure	Comments	Reference
Weight gain, survival, in SD rats, urinalysis, hematology, blood biochemistry after exposure	849 MHz CDMA and 1.95 GHz WCDMA, 45 min/day, 5 days/week for 1 year, at 2 W/kg per signal, animals freely moving, am or pm alternately	No significant effects except significant increase in mean corpuscular hemoglobin level and alkaline phosphatase in males, and significant decrease in total bilirubin and lactate dehydrogenase in females		(48)
Weight gain, survival, lymphoma incidence in AKR/J mice, blood biochemistry after 26 weeks of exposure, metastatic infiltration by IHC	849 MHz CDMA and 1.95 GHz WCDMA, 45 min/day, 5 days/week for 42 weeks, at 2 W/kg per signal, animals freely moving, am or pm alternately	No significant effects. Significant decrease in metastatic infiltration in brain of males, increase in females, not due to exposure		(49)

*SD, Sprague-Dawley; CDMA, Code Division Multiple Access; WCDMA, Wideband CDMA; IHC, immunohistochemistry*.

*Specific energy absorption rate values are mean whole-body averages, significant indicates statistical significance*.

In one study, young rats were exposed to CDMA and WCDMA signals for 45 min/day, 5 day/week for a year (48). Animals were exposed alternately in the morning or afternoon. No significant effects on weight or on spontaneous tumor rates were found, and post-mortem analysis did not show any significant pathological differences that could be related to exposure. In addition, analysis of blood and urine did not reveal any significant field-related effects except for a significant increase in mean corpuscular hemoglobin level, and alkaline phosphatase in males; and a significant decrease in total bilirubin, and lactate dehydrogenase in females.

In the other study, young AKR/J mice (which express the ecotropic retrovirus AKV in all tissues and spontaneously develop lymphoma) were exposed to combined CDMA and WCDMA signals for 45 min/day, 5 day/week for 42 weeks (49). Compared to sham-exposed controls, exposure had no significant effect on weight, survival time, or incidence of lymphoma. The latter was assessed by histopathological analysis of the thymus. Blood counts remained unaffected by exposure and there were no consistent effects on metastatic infiltration in the spleen or other organs (changes in infiltration seen in the brain were attributed to other factors, not exposure). In addition, but not related to carcinogenesis, the same research group has also reported that combined exposure to 848 MHz CDMA and 1950 MHz WCDMA signals have no teratological effect on ICR mice exposed throughout gestation (50); no effect on testicular function in SD rats exposed for 12 weeks (51); no effect on immune functions in male SD rats exposed for 8 weeks (52); nor any effect on endocrine function in male and female SD rats exposed for 4 or 8 weeks (53). Animals in these four studies were exposed for 45 or 90 min/day for 5 days/week at a total whole-body average SAR of 4 W/kg.

Another research group has examined the effects of a simultaneous exposure to 900 MHz and 2.45 GHz fields on endpoints that are relevant to carcinogenesis but did not investigate malignant pathology. In these studies (not included in Table 8), restrained male rats were exposed at up to 0.2 W/kg in a GTEM cell, and SARs were estimated using numerical phantoms. In the first study, it was reported that single or simultaneous exposure had no effect on cellular morphology and apoptosis in eight tissues, including the brain, muscle, and testicles (54). In the second study, effects on cellular stress responses were investigated in the cerebral cortex and cerebellum (55). Some significant changes were seen in heat shock protein and caspase-3 expression, but simultaneous exposure did not result in any consistent effect compared to either single frequency alone. The average whole-body SAR of the combined signal was comparable, if slightly less than the value for the single frequencies (approximately 0.03–0.08 and 0.05–0.09 W/kg respectively). The authors concluded that simultaneous exposure to two low-level signals at different frequencies did not result in any additive effects or changes that were greater than those of each signal separately.

In summary, high-quality experimental studies with animals (and mechanistic *in vitro* studies) should be informative and help to decide whether RF fields can have a significant carcinogenic effect. The available evidence from animal studies does not suggest long-term, low-level exposure to either a single or two RF fields can have a significant influence on carcinogenesis. The majority of well-performed studies using a single frequency provide strong evidence for an absence of effects, particularly for tumors of the brain and nervous system, with no consistent changes reported on weight gain, survival, or increases in any tumor type or non-plastic lesion seen. In addition, no consistent dose–response trends have been reported in any study. Two studies using the same mouse model, however, report long-term exposure at whole-body SARs as low as 0.04 W/kg may increase the potential of a chemical mutagen to cause tumors, and preliminary results from an NTP study suggest increased risks for at least two tumor types after lifetime exposure of male rats to 900 MHz at a few W/kg. There are far fewer studies with animals simultaneously exposed to more than one frequency, and the available evidence is in line with the data from the single frequency studies, and no consistent effects of exposure have been seen.

## Future Research Needs

Members of the public are exposed to a complex and variable mixture of RF (and lower) frequency electromagnetic fields and exposures critically depend on the distance from the source, together with the emitted power and duty factor. Mobile phones, which tend to be the major source of RF exposure, have adaptive power control to adjust their output power to preserve battery life, which further complicates personal exposure assessment. By contrast, animal studies have used at most two RF signals, and usually at constant power, although some studies have increased their relevance to the human situation by using a signal that mimics the output of mobile phone during a conversation. Overall, the fields used in animal experiments are not typical of all the fields that are experienced by mobile phone users in everyday life, although generally represent the dominant near-field exposure.

Therefore, it might be argued that further research with animals would be needed in order to clarify the relevance of combined, multiple fields to cancer risk under realistic exposure conditions. However, such a programme of work is considered unfeasible at this time, and is not recommended as a high priority for the following reasons.

First, neither the animal or human data suggest that substantial increased risks are associated with exposure to RF fields under any particular circumstances. The animal data have investigated a variety of models and none has produced unequivocal evidence of increased risks, even with long-term and intense exposures, and using models that are considered more sensitive to reveal effects. Similarly, some of the human data are suggestive of increased risks, but overall fail to provide conclusive proof of harm. Both lines of research have different strengths and weaknesses, but generally they tend to complement and inform one another, and reinforce their findings. Thus, there is no overwhelming suggestion from existing observational or experimental studies that exposure of animals to multiple fields is likely to yield more positive, field-related results (although if such studies were performed and they produced negative effects, that data would still be of interest and provide additional inputs to risk assessment of these fields).

Another reason for this recommendation is a lack of plausible interaction mechanism whereby two or more RF signals could interact to produce a more than additive effect. Presently, the absorption of power leading to tissue heating is the main mechanism whereby RF fields have an effect on living tissues (stimulation of excitable tissues by induced electric fields at up to 10 MHz is the other established mechanism). In the case of heating, the combined effect of exposure is the sum of the absorbed powers (56). Different frequencies would have different penetration into the body leading to different patterns of absorption, but the time-averaged thermal burden is considered to be the sum of the individual heating components. Thus, it is not possible to envisage exposure to multiple weak fields as having a greater overall effect than a single field at that combined intensity. Of course, this does not deny that if exposures induce hyperthermia then circulatory and other changes will occur (57) and if exposure is sufficiently intense or prolonged this can result in thermal damage to tissues, but even in this context, hyperthermia from RF fields is not considered to be carcinogenic (58) so even intense exposures should not increase the risk of cancer.

In addition, although it has sometimes been suggested that specific frequencies, modulations, or pulse shapes can be more effective at producing biological effects than continuous wave or sinusoidal fields (59, 60) such a proposition has not been firmly established and the results of the animal cancer studies (as described in Tables 3–8) do not suggest the existence of any field- or modulation-related effects, and exposure to any particular signal never consistently resulted in any field-related effects. Furthermore, attempts to identify such a non-thermal mechanism that could operate at all RF frequencies and realistic field intensities have not proved successful [see 3 (61),]. A systematic approach to the effects literature identified five credible non-thermal mechanisms (62). These included iron-ion-mediated reactions, and the radical pair mechanism. However, selective microthermal heating was considered the most plausible mechanism, although it lacked experimental verification of the microthermal properties of cells. In another detailed review of potential mechanisms, it was concluded that the dominant mechanism at RF frequencies was dielectric heating (63). Most other putative mechanisms were considered implausible as a means for independent energy deposition because they would result in temperature rises that would overwhelm any other biological response. Resonant molecular or sub-molecular vibrational modes were also excluded because they would be too heavily damped, while other mechanisms involved energy that would be far weaker than the thermal background. It was also concluded that field strengths that are greatly in excess of those that would cause dielectric heating would be necessary to interact directly with charges or dipoles. However, it was suggested that non-linear processes such as rectification could transduce frequency-modulated signals into a more biologically relevant frequency range where physiological systems operate. In a direct test of this suggestion, a doubly resonant cavity was used to search for non-linear energy conversion in a wide range of biological samples that included cancer cells and slices of mouse tissues (64). Samples were exposed to a continuous wave field at the resonant frequency of the cavity and monitored for generation of the second harmonic. The absence of any consistent second harmonic indicated a lack of support for the hypothesis, since second harmonic generation was considered to be a necessary and sufficient condition for demodulation. Beyond a gradual change from effects based on induced electric fields to ones based on heating with increasing frequency, the possibility of explicit effects at specific frequencies are also absent in scientifically based guidelines limiting human exposure to RF fields (56, 65). Overall, while it cannot be completely dismissed, it seems increasingly less likely that there could be some previously unidentified non-thermal mechanism that only operates with exposure to some specific combinations of fields or modulations.

In the absence of a clear hypothesis to guide the choice of the particular frequencies to be investigated, nor any specific information on the important exposure parameters, such as intensity, duration, or pattern of exposure, it is reasonable to adopt a pragmatic approach and use the two or three fields that dominate personal exposure, perhaps in terms of maximum incident power density or time-averaged whole-body or local SAR, but even this approach has no absolute guarantee of success. It would be extremely expensive and unrealistically time-consuming to try all the various combinations of fields to which people are commonly exposed in order to discover the most effective combination. Such an approach is far better suited to high-throughput *in vitro* techniques (66) and not long-term studies with animals.

Finally, it would be absolutely essential to decide on an appropriate biological model to use in these studies. The use of different mouse models for investigating (ionizing) radiation-induced cancers has been reviewed (67). It was suggested that the ideal mouse model possesses a low spontaneous background frequency of the desired malignancy, has a short latency period, avoids the co-development of cancers at alternative sites, and produces nearly identical tumors to the corresponding human cancer in terms of onset, progression, and underlying pathology (67). But it is acknowledged that a perfect model may not exist, so the best available model would have to be used depending on the type of cancer being investigated. One possible mouse to use to investigate brain cancer might be the *Patched1* (*Ptc1*^+/−^) knockout model. This is a well-established model for carcinogenesis, and was previously used to investigate effects of RF fields (30). One practical advantage of this model is that since sensitivity to X-irradiation is known to be greatest in early postnatal life in this model, exposure to multiple RF fields could be restricted to the period before weaning, and the tumor yield measured and characterized in response to exposure. Other suitable models would have to be identified for other types of cancer. As with previous animal studies, these studies would not be trying to replicate use of mobile phones *per se*, but to provide information about qualitative effects of exposure to RF fields. Mouse models cannot generally provide reliable estimates of risk in humans due to inherent differences in anatomy and body size, and in metabolism and lifespan. Differences also exist in DNA repair capacity, and in the etiology of tumor development (68). Overall, based on this analysis, the likelihood that exposure to multiple, low-level RF exposures would have unexpected consequences and significantly increased the risk of any type of cancer is considered to be low, but the possibility cannot be completely excluded. Nevertheless, it would be prudent to initiate such studies with animals only once an interaction mechanism had been better identified, and at least some of the more pertinent exposure parameters were known.

## Conclusion

Most long-term animal studies investigating the carcinogenic potential of RF fields have used a single frequency, usually at one intensity, which is in contrast to the variable combination of fields and intensities that may be experienced by people in the everyday environment. These studies suggest that long-term exposure of animals is unlikely to affect the initiation or development of any type of cancer, but this possibility cannot be dismissed completely as a few studies have reported field-related changes.

Thus, it might seem sensible to consider the need for additional animal studies using exposures that are more typical of the multiple sources that are likely to be encountered by a member of the public (as defined in Tables 1 and 2), especially given the widespread and increasing use of wireless and other technologies. However, as exposures to multiple weak fields are only known to have no greater effect than a single field at the combined intensity, such research should only be undertaken if it is considered plausible that there could be an as yet unknown biological interaction mechanism whereby multiple fields could pose an increased carcinogenic hazard. Knowledge of this mechanism might also help to guide selection of the fields to be tested, as otherwise there is little explicit information to suggest which frequencies to use beyond the dominant exposure from use of a mobile phone. In the absence of a possible mechanism and uncertainty about the signals, additional work with animals exposed to multiple RFs is considered unlikely to yield valuable information about field-dependent effects (although data showing no exposure-related effects can still be useful for risk assessment). Therefore, animal studies investigating the carcinogenic potential of exposure to multiple RF frequencies should not be given a high priority for research at this time.

## Author Contributions

ZS conceived the work, and with KAB, AG, and LL contributed equally to the interpretation and drafting on the biology. CC, DA, and MM contributed equally in the acquisition of the measurement data and drafted the sections on measurements. All authors approved the final version and agreed to be accountable for all aspects of the work.

## Conflict of Interest Statement

The authors declare that this work was conducted in the absence of any commercial or financial relationships that could be constructed as a potential conflict of interest, except Sienkiewicz declares that he has owned 440 ordinary shares in BT Group, a communication services company.
